# An on‐premise study to investigate the effects of mixing alcohol with caffeinated beverages

**DOI:** 10.1002/brb3.2445

**Published:** 2022-02-08

**Authors:** Sean J. Johnson, Joris C. Verster, Chris Alford

**Affiliations:** ^1^ Centre for Trials Research Cardiff University Cardiff CF14 4YS UK; ^2^ Psychological Sciences Research Group University of the West of England Bristol UK; ^3^ Division of Pharmacology Utrecht Institute for Pharmaceutical Sciences (UIPS) Utrecht University Utrecht The Netherlands; ^4^ Centre for Human Psychopharmacology Swinburne University Melbourne Australia

**Keywords:** alcohol consumption, alcohol, AMED, caffeine, energy drink

## Abstract

**Objective:**

The purpose of this on‐premise study was to determine if mixing alcohol with caffeinated mixers had an impact on objective and subjective intoxication.

**Methods:**

The study was conducted across eight drinking occasions in the City of Bristol, UK. Participants (*N* = 1041) were recruited outside popular night‐time entertainment venues and interviewed regarding their alcohol consumption for that particular evening, including whether or not they had consumed caffeinated beverages with alcohol. Subjective intoxication was rated on an 11‐point scale and objective intoxication determined with a breath alcohol test. Depending on their consumption on the night of the interview, participants also reported whether they consumed alcohol mixed with caffeinated mixers or alcohol‐only on other consumption occasions.

**Results:**

Between‐subjects analyses found that alcohol–caffeine consumers consumed more alcohol and had higher objective and subjective intoxication than those who consumed alcohol‐only. These results remained significant regardless of whether or not they mixed alcohol with caffeinated mixers or consumed alcohol‐only on the night of the interview. Within‐subject analyses revealed that alcohol–caffeine consumers drank the same or less alcohol on alcohol–caffeine occasions compared to alcohol‐only occasions.

**Conclusions:**

These findings provide support that alcohol–caffeine use does not increase overall alcohol consumption, and may be one manifestation of a high risk‐taking personality.

## Graphical abstract

This on‐premise study found that caffeine‐alcohol use does not increase overall alcohol consumption per se but that caffeine‐alcohol use may be one manifestation of a high‐risk taking personality.

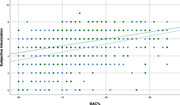

## 1 INTRODUCTION

Excessive alcohol consumption is an internationally accepted public health challenge with wide‐ranging social, health, and economic consequences. Binge drinking, defined as consuming large amounts of alcohol in a short space of time or drinking to get drunk (National Health Service, 2019), is particularly problematic and prevalent among adolescents and young adults. While 16–24 year olds are less likely to have drunk alcohol in the past week compared to older age groups, when they do drink, they are more likely to drink at high levels (Office for National Statistics, 2018). Given the extent of this problem, in recent years, much research has been conducted to try to understand the factors that might be driving this excessive alcohol consumption.

One factor that has been linked to problematic alcohol consumption among this age group is the rise in popularity of alcohol mixed with energy drinks (AMED). Indeed, despite energy drinks comprising only 5% of the total soft drinks market (British Soft Drinks Association, 2017), 39% of UK students aged 18–30 years old report consuming AMED at least once in the past month (Johnson et al., 2016), and higher than percentages observed in some other countries (Vida & Racz, 2015). Therefore, it appears to be a particularly popular consumption practice and intended use for energy drinks among UK young adults, although representing only around 5% of their total caffeine intake (Morris & Elgar, 2020).

Given the popularity of AMED, and that caffeine is considered the principle ingredient for energy drink effects (Committee on Toxicity in Food, 2018; EFSA Panel on Dietetic Products, 2015), it is logical for questions to be raised regarding its interaction with alcohol (Alford et al., 2012). Research has consistently demonstrated that energy drinks have the ability to improve performance and increase alertness and stimulation (Alford et al., 2001; Kennedy & Scholey, 2004; Scholey & Kennedy, 2004; Seidl et al., 2000; Warburton et al., 2001), whereas alcohol typically impairs performance and increases sedation (Julien et al., 2011; Sukhes et al., 2008). Based on the individual pharmacodynamic and behavioral effects of these beverages, it has reasonably been theorized that their combined use results in the stimulant effects of caffeine in energy drinks counteracting the sedative effects of alcohol, thus reducing physiological and psychological sedation while increasing stimulation (Arria & O'Brien, 2011; Peacock et al., 2014). A suggested mechanism of action is that coadministration may attenuate the sedative effects of alcohol by blocking alcohol‐related increases in extracellular adenosine and increase the stimulant effects of alcohol by potentiating alcohol‐induced dopamine release via the A2A‐D2 receptor heteromer (Ferré, 2016; Ferré & O'Brien, 2011). If such an effect does exist, AMED consumers may feel less impaired and less intoxicated than they actually are, but potentially with the functional impairment of alcohol remaining.

On this basis, some academics (Arria & O'Brien, 2011; Pennay et al., 2011) and health organizations (National Health Service, 2017) have expressed concern regarding the potential risks associated with AMED consumption. These may include consumers perceiving themselves as less intoxicated and able to drive when they are objectively above the drink drive limit, or continuing to drink further quantities of alcohol despite already being intoxicated leaving themselves susceptible to negative consequences, such as getting into fights or having unprotected sex. Given these potential negative outcomes, it is important to objectively determine whether the coconsumption of alcohol with caffeine, via mixers such as colas or energy drinks, results in undesirable functional consequences.

A meta‐analysis revealed that these concerns are not supported by scientific data (Benson et al., 2014). Consistently, across experimental studies, subjective intoxication after fixed amounts of alcohol revealed no significant differences between alcohol alone and alcohol plus caffeine. However, in these controlled laboratory‐based experiments alcohol intake was modest and standardized, and achieved blood alcohol concentration (BAC) of 0.032%–0.12%, which is lower than BACs reported in real‐life drinking in naturalistic studies or assessed in on‐premise studies. Early survey‐based research (O'Brien et al., 2008) that found AMED consumers drank significantly more alcohol and engaged in more alcohol‐related harms than alcohol‐only (AO) consumers (between‐subjects) was used to develop the AMED masking theory. This finding has been consistently demonstrated by other researchers (Brache & Stockwell, 2011; de Haan et al., 2012; Eckschmidt et al., 2013; Johnson et al., 2016; Lubman et al., 2013; Trapp et al., 2013; Woolsey et al., 2010; Woolsey, Jacobson, et al., 2015; Woolsey, Williams et al., 2015). However, it has also been shown that AMED consumers differ in a range of characteristics from AO consumers including increased sensation seeking and risk‐taking (Verster et al., 2018). Therefore, an alternative explanation may be that AMED consumption is one of a cluster of behaviors expressed by some underlying trait or phenotype (Verster et al., 2012). This has been supported by within‐subject research that controls for phenotypical differences that have found no difference or a reduction in the amount of alcohol consumed by AMED consumers on occasions they drink AMED compared to the occasions they consume AO (de Haan et al., 2012; Johnson et al., 2016; Lubman et al., 2013; Newcombe et al., 2019; Woolsey et al., 2010). Although three studies have shown an increase in alcohol consumption during AMED occasions (Brache & Stockwell, 2011; Peacock et al., 2012; Price et al., 2010).

In summarizing this research, a recent systematic review and meta‐analysis (Verster et al., 2018) concluded that while AMED consumers drank significantly more alcohol than AO consumers (between‐subjects analysis), among AMED consumers (within‐subject analysis) alcohol consumption did not significantly differ between AMED and AO occasions. In addition, the meta‐analysis also found no significant masking‐effect in the experimental studies that directly compared subjective intoxication after consuming AMED with AO. These findings are in contrast to what would be expected if AMED was causal in increasing overall alcohol consumption.

However, the previous studies upon which this conclusion is based have several limitations. First, survey research is reliant on self‐reported previous consumption, often of a “typical” or “heaviest” drinking occasion. The ability to accurately recall the number and type of drinks consumed is likely to be affected by the time frame passed and alcohol‐related amnesic effects (Platt et al., 2016; Verster et al., 2003). Second, in experimental studies ethical approval limits the amount of alcohol and caffeine that can be administered. For example, in the studies that compared AMED to AO the typical amount of alcohol and caffeine consumed was the equivalent of one to four alcoholic drinks and half to three 250 ml cans of energy drink respectively (Verster et al., 2018). However, as evident in survey research (Johnson et al., 2016) these levels may be significantly lower than those observed in the UK night‐time economy. In addition, laboratory studies are unable to fully replicate the real‐world context in which consumption takes place (Verster et al., 2019) that might influence the association between energy drink consumption and intoxication. Therefore, it is important to examine the possibility of an AMED masking effect in real‐world drinking occasions.

Recently, a number of on‐premise studies have been conducted to investigate the effect of AMED consumption. These typically involve approaching participants during or immediately after their drinking session and asking them to report on their alcohol consumption that evening and provide an objective measure of intoxication via a breathalyzer. These studies have typically found that AMED consumers have significantly higher BAC readings (Devilly et al., 2017; Lubman et al., 2014; Lubman et al., 2013; Miller et al., 2013; Pennay et al., 2015b; Thombs et al., 2009) and subjective intoxication scores (Lubman et al., 2014; Miller et al., 2013) than those who consumed AO.

However, given the lower frequency of AMED drinking occasions versus AO drinking occasions among AMED consumers (Peacock et al., 2015), it is likely that in the above studies AMED consumers were contained within those categorized as AO consumers, but they had not consumed AMED on the night of the interview. Verster et al. (2015) asked participants about their alcohol consumption with and without energy drinks, for that particular evening and for other occasions. They found that those who reported consuming AMED that evening did not significantly differ in both subjective and objective intoxication compared to those who reported consuming AMED on other nights and those who never consumed AMED (either that night or on other occasions). Whether or not participants consumed energy drinks did not predict subjective intoxication.

A further aspect to consider is that, in line with survey research, the majority of on‐premise studies have utilized between‐subjects comparisons in reaching cause–effect explanations for the impact of energy drinks on alcohol consumption. As previously outlined, this approach does not control for the many phenotypical differences between consumption groups that may explain increased alcohol consumption. Within‐subject comparisons by Verster et al. (2015) revealed that among AMED consumers, there were no significant differences in total alcohol consumption between AMED occasions and AO occasions. This study suggests that at real world consumption levels mixing alcohol with energy drinks does not increase overall alcohol consumption or mask subjective intoxication.

Given that caffeine is believed to be the main ingredient in energy drinks to cause a purported masking effect, it is also important to investigate the effects of other popular caffeinated beverages, such as cola, on objective and subjective intoxication. Indeed, many researchers have recently questioned whether energy drinks are unique caffeinated mixers in their effects on alcohol consumption (Cobb et al., 2015; Johnson et al., 2018; Thombs et al., 2010).

The only previous on‐premise study to examine alcohol consumption when mixed with other caffeinated beverages found that, while those who consumed AO were less intoxicated, there were no significant differences in the intoxication levels of those who mixed alcohol with cola beverages and those who mixed alcohol with energy drinks (Thombs et al., 2010). However, this study only presented between‐subjects comparisons and did not assess subjective intoxication and so was unable to determine whether alcohol mixed with other caffeinated beverages (AOCM) had a differential effect on objective and subjective intoxication compared to consuming AMED or AO.

Given the limitations of previous research, the present on‐premise study aims to examine whether there are any differences in alcohol consumption practices between those who consume AO and those who consume AMED, AOCM or both (between‐subjects). In addition, in order to investigate whether caffeine consumption has an impact on overall alcohol consumption, drinking occasions on which participants consumed AO will be compared with other occasions on which they consumed AMED and AOCM (within‐subject).

## 2 METHODS

### 2.1 Setting

The on‐premise study took place in the City of Bristol, United Kingdom. Bristol is the largest city in the South West of England, and one of the ten “core cities” in the United Kingdom, with a population of 463,400 people (Bristol City Council, 2020). The city's cosmopolitan culture has a vibrant university population, made up of the University of Bristol (population around 22,000) and the University of the West of England (population around 29,000), with many popular and busy entertainment districts with late opening hours. The legal drinking age in the United Kingdom is 18 years and the legal BAC limit for driving in England is 0.08%.

The study was undertaken outside night‐time entertainment venues identified as popular during observational visits. Given that most alcohol‐related incidents occur in close proximity to night‐time entertainment venues (Allen et al., 2003) alcohol consumers were assessed as they exited the premises. It was hoped that this approach would provide a representative view of the typical alcohol consumption practices faced by the City of Bristol night‐time economy. Prior to the study commencing venue owners and local police were contacted and informed of the study aims and data collection period. Data was collected over a two‐week period on a Tuesday, Thursday, Friday, and Saturday night, the most popularly attended nights out in the City of Bristol. Discussion with night‐time entertainment venues allowed the research team to ensure they were present at appropriate times to optimize data collection as potential participants left premises. This was between 10 pm and 3 am on Tuesday and Thursdays, and between 11 pm and 4 am on Friday and Saturday.

### 2.2 Procedure

Given that participants will have consumed alcohol and are likely to be keen to find food outlets and secure transport home after their night out, it was important to keep data collection time to a minimum. Therefore, the on‐premise study comprised of a short survey and a breathalyzer test and took approximately 5 min to administer. The current methodology was similar to that successfully employed by Verster et al. (2015).

Twelve trained research assistants were split into six teams of two testers. Where possible this included one male and one female researcher. This was to facilitate engagement with potential participants from the same and opposite sex, as well as providing an additional level of safety. The principle investigator oversaw all study nights and was responsible for liaising with venue staff, ensuring safety and conducting quality control checks of procedures. All team members wore hi‐visibility clothing labeled “Alcohol Researcher” and carried an alarm that emitted a loud bleeping noise to attract attention if they required assistance.

Potential participants were selected as they were leaving premises, on adjacent walkways, taxi ranks, and bus stops. Research assistants were trained to approach a mixture of gender, ages, and students/nonstudents in order to reflect the demographic of Bristol City night‐time patrons. As soon as one interview had finished, the researchers approached the next potential participant. Once approached, the purpose of the study was explained to potential participants via an information sheet and informed consent obtained. Research assistants were trained to implement the recommendations by Aldridge and Charles (2008) that have been extensively used in previous on‐premise studies that acknowledge intoxication in the informed consent process. One researcher conducted the survey while the other prepared to conduct the breathalyzer test.

To attract participants and decrease nonresponse bias, participants were given the option to enter into a monetary prize draw upon completion of the study. As used successfully in the previous survey research in the United Kingdom (Johnson et al., 2016) the monetary prizes were 1 × £500 and 10 × £50.

### 2.3 Survey content

Given the short time frame to engage with potential participants, only a few but key questions were asked. Demographics were limited to sex, age and student status of participant. All participants were asked to report the number of alcohol units consumed that evening and the period of time over which they consumed them. This was split into the number of alcohol units and time spent consuming alcohol prior to going out (predrinking) and the number of alcohol units and time spent consuming during the night out (on‐premise). To assist in accurately describing their alcohol intake participants were provided with picture and written examples of how many units of alcohol are in commonly consumed beverages. An alcohol unit in the United Kingdom is defined as 8 g/10 ml of pure alcohol = 1 unit (Drinkaware, 2020). The research assistants assisted participants in calculating the alcohol units where required. Participants were also asked to report any smoking or drug use that evening, but this was not objectively tested. Given that participants were unlikely to be familiar with BAC measurements and units, participants were asked to estimate their perceived level of intoxication on a visual analogue 11‐point scale ranging from 0 (sober) to 10 (highly drunk). Subjective intoxication was asked prior to the breathalyzer test to ensure that their result did not influence them when estimating their level of intoxication. Participants then completed the 3‐item Alcohol Use Disorders Identification Test (AUDIT‐C) (Bush et al., 1998), to identify potential risk for alcohol use disorder. The score on the AUDIT‐C ranged from 0 to 12, with a score of 5–7 indicating increased risk, 8–10 higher risk, and 11–12 possible alcohol dependence.

Following this, participants were asked whether they had mixed alcohol with caffeinated beverages (i.e., energy drinks, cola) that evening. If yes, participants were asked to report the type and quantity of caffeinated beverages consumed prior to going out (predrinking) and during the night out (on‐premise). To assist in accurately describing their intake of caffeinated beverages, participants were provided with picture examples of typically consumed beverages. One standard caffeinated beverage = 80 mg caffeine.

Depending on their consumption on the night of the interview, participants were then asked to indicate whether they consumed alcohol mixed with caffeinated mixers or alcohol only on other consumption occasions and the amount of alcohol they usually consumed.

### 2.4 Breath alcohol analysis

In order to objectively measure the amount of alcohol consumed, participants were asked to complete a breath alcohol test using an Alkohit X100 breath analyzer. This device used the UK standardized blood to breath ratio (BBR) of 2300:1 to calculate Blood Alcohol Concentration (BAC) and provided accurate readings ranging from 0.00% to 6.30% BAC with a measuring accuracy ± 5% BAC when calibrated. Prior to conducting the breath test participants were asked if they had consumed alcohol or smoked cigarettes in the preceding 15 min, and if so asked to wait until this minimum time had passed.

To conclude the on‐premise interview participants were informed of their BAC reading and asked whether they planned on continuing drinking after the interview or planned to go home. If they answered that they planned on going home, they were asked their mode of transport. If they planned on driving themselves, either by car, motorbike or cycle, depending on the BAC measurement they were advised not to do so and to take a taxi instead. The researchers also offered to arrange a taxi for the participant if required. The study protocol was reviewed and approved by the University of the West of England Research Ethics committee (Approval number: HAS.16.03.117).

### 2.5 Sample

A total of 1041 potential participants engaged with the research team and provided informed consent. Twenty‐seven participants decided to withdraw prior to completing the on‐premise survey and 30 provided insufficient information to be included in the sample. Those reporting drug and medication use were also excluded (*N* = 62). A small proportion of participants reported mixing alcohol with caffeinated mixers on the night of the interview but not consuming AO on other drinking occasions (*N* = 29). On the night of the interview 13 of these participants reported consuming AOCM, 11 reported consuming AMED, and 5 reported consuming AMED & AOCM. Given their low frequency and lack of comparison with AO drinking occasions, these participants were excluded from the current analysis. After data cleaning, a total of 148 participants were excluded, giving a complete dataset of 893 responses that were entered into the analysis.

Participants ranged between 18 and 50 years old, with a mean (SD) age of 22.7 years (± 4.0), and just over half of the study sample were female (*N* = 453, 50.7%). One in four participants reported smoking that evening (26.9%), with an average of 8.0 (± 3.5) cigarettes consumed. Predrinking was reported by 65.5% of respondents with a mean of 2.9 (± 3.0) alcohol units consumed before going out, a mean of 5.7 (± 3.1) alcohol units consumed while on‐premise, and a mean total of 8.6 (± 4.2) alcohol units consumed during the entire drinking occasion. Participants recorded a mean BAC of 0.12% (± 0.07%), and a mean subjective intoxication (0–10) score of 4.8 (± 1.9). The average total AUDIT‐C (0–12) score was 7.2 (± 2.0).

On the night of the interview 73.6% (*N* = 657) of the sample reported consuming AO, 11.9% (*n* = 106) reported consuming AOCM, 9.4% (*N* = 84) reported consuming AMED, and 5.2% (*n* = 46) reported consuming both AMED and AOCM. When asked to indicate their alcohol consumption patterns on other drinking occasions four consumer types were identified: AO consumer (*N* = 291, 32.6%), AMED consumer (*N* = 128, 14.3%), AOCM consumer (*N* = 135, 15.1%), and mixed consumer (*N* = 339, 38.0%). A combination of consumer type and consumption on the night of the interview defined the between‐subjects groups for analysis (Table 1).

**TABLE 1:** Participant groups based on consumer type and consumption on the night of the interview

		Consumption on other drinking occasions		
Consumer type	Consumption on night of interview	AMED?	AOCM?	AO?
AO consumer (291)	AO (291)	✗	✗	–
AMED consumer (128)	AO (85)	✓	✗	–
	AMED (43)	–	✗	✓
AOCM consumer (135)	AO (86)	✗	✓	–
	AOCM (49)	✗	–	✓
Mixed consumer (339)	AO (195)	✓	✓	–
	AMED (41)	–	✓	✓
	AOCM (57)	✓	–	✓
	AMED & AOCM (46)	✓	✓	✓

**Abbreviations**: AO, alcohol only; AMED, alcohol mixed with energy drinks; AOCM, alcohol mixed with other caffeinated mixer; ✗, not consumed; ✓, consumed; –, not applicable.

### 2.6 Data collection and statistical analysis

During the on‐premise study, data were collected by the research assistant using paper and pen rested on a clipboard. Data were then entered and analyzed using IBM SPSS Version 26 (IBM SPSS Statistics 2019 Armonk, NY, USA). The mean, standard deviation, and frequency distributions were calculated for all variables.

Between‐subjects comparisons were conducted at two levels. First, participants were grouped into consumer types depending on whether or not they had consumed AO, AMED, or AOCM either on the night of the interview or on other drinking occasions. This allowed an investigation into whether there were any underlying differences (age, gender, alcohol consumption practices) between consumer types regardless of what they had consumed on the night of the interview. Second, these consumer types were further differentiated by their alcohol consumption on the night of the interview to determine if there was a pharmacological effect of adding caffeine to alcohol.

For the between‐subjects comparisons, data were analyzed using analysis of variance (ANOVA). Post hoc Tukey‐HSD corrected for multiple comparisons. Where the assumption of equal variance using Levene's test of homogeneity of variance could not be assumed Welch's *F*‐test was performed and where appropriate Games–Howell post hoc used. Chi‐square tests were used to analyze categorical data.

In order to investigate the association between objective and subjective intoxication and the possibility of a masking effect Pearson's correlations were computed comparing those who consumed AO on the night of the interview, with those who consumed AMED or alcohol with caffeine in any form (AMED‐tonight, AOCM‐tonight, AMED & AOCM‐tonight combined). In addition, differences between these groups at different BAC ranges were investigated using an independent samples *t*‐test.

For the within‐subject comparisons consumer types were analyzed using paired samples *t*‐test comparing total alcohol consumption on occasions when individuals do and do not combine alcohol with energy drinks (AMED consumers) or other caffeinated mixers (AOCM consumers). In addition, a repeated measures analysis of variance with Huynh–Feldt correction was used to compare total alcohol consumptions on AO, AMED, and AOCM occasions among the mixed consumer group. Differences within‐subject were explored using post hoc tests with Bonferroni correction.

All tests were two tailed and differences were regarded as significant at *p* < .05. Appropriate effect sizes are reported for all significant findings (Lenhard & Lenhard, 2016).

## 3 RESULTS

### 3.1 Between‐subjects comparisons

#### 3.1.1 Consumer type

To investigate whether underlying differences exist between those that consume AO and those who mix alcohol with different caffeinated beverages (AMED and AOCM), between‐subjects analyses were performed on demographic and alcohol consumption practices between consumer types (see Table 1).

There was a significant main effect of consumer type on age (*F*(3, 377) = 17.81, *p* ≤ .001, *η* ^2^p = .023) with participants from the AO consumer type being significantly older when compared to the AMED and mixed consumer types, but not the AOCM consumer type. The AO consumer type scored significantly lower (*F*(3, 354) = 52.87, *p* ≤ .001, *η* ^2^p = .039) on the AUDIT‐C when compared to all the alcohol–caffeine consumer types (AMED, AOCM, and mixed consumer types). There were no significant differences in gender (χ^2^(3, *N* = 893) = 3.79, *p* = .285), student status (X^2^(3, *N* = 893) = 5.61, *p* = .132), the percentage of smokers (χ^2^(3, *N* = 893) = 2.56, *p* = .465), or the number of cigarettes consumed on the night of the interview (*F*(3, 88) = 1.41, *p* = .244) between any of the consumer types. See Table 2 for group means and standard deviations.

**TABLE 2:** Between‐subjects comparison of consumer types regardless of consumption on night of the interview

	AO consumers (*n* = 291)	AMED consumers (*n* = 128)	AOCM consumers (*n* = 135)	Mixed consumers (*n* = 339)
Age (years)	24.0 (4.1)	21.7 (2.6) [0.62]^a^	22.8 (4.7)	22.0 (3.8) [0.51]^a^
Gender (% female)	53.5 (± 5.7)	52.3 (± 8.6)	54.1 (± 8.4)	46.6 (± 5.3)
Student status (% students)	53.6 (± 5.7)	54.7 (± 8.6)	60.0 (± 8.3)	62.2 (± 5.2)
BAC%	0.09 (0.06)	0.14 (0.08) [0.75]^a^	0.14 (0.08) [0.75]^a^	0.12 (0.07) [0.46]^a^
Alcohol units predrinking	2.4 (2.8)	3.4 (3.2) [0.34]^a^	3.0 (2.5)	3.2 (3.2) [0.27]^a^
Alcohol units on‐premise	4.4 (2.3)	6.2 (3.3) [0.68]^a^	6.3 (3.6) [0.68]^a^	6.2 (3.1) [0.65]^a^
Total alcohol units	6.9 (3.4)	9.7 (4.8) [0.72]^a^	9.3 (3.8) [0.68]^a^	9.4 (4.3) [0.64]^a^
Subjective intoxication score	4.1 (1.7)	5.2 (2.0) [0.61]^a^	5.0 (2.0) [0.50]^a^	5.2 (1.9) [0.61]^a^
Total time predrinking	2:14 (1:31)	2:18 (1:31)	2:25 (1:38)	2:33 (1:47)
Total time on‐premise	3:36 (1:53)	3:22 (1:56)	3:28 (1:38)	3:22 (2:03)
Total drinking time	4:56 (2:28)	4:59 (2:22)	5:16 (2:10)	4:58 (2:25)
Total AUDIT‐C score	6.2 (1.5)	7.8 (1.9) [0.98]^a^	7.4 (1.8) [0.75]^a^	7.8 (2.0) [0.90]^a^
Smoking (% yes)	23.7 (± 4.9)	30.5 (± 7.9)	28.1 (± 7.5)	27.7 (± 4.8)
Total number of cigarettes smoked	7.2 (4.1)	8.3 (2.0)	8.8 (4.2)	8.1 (3.5)

***Notes***: Mean and standard deviation (SD, between brackets) or percentages and 95% confidence interval (between brackets) are shown. Hedge's *g* effect size [between square brackets] where appropriate.

^a^ Significantly different (*p* < .05) to the AO consumer group. Subjective intoxication scale = 0–10, time = hours: minutes, AUDIT‐C score = 0–12, one alcohol unit = 8 g/10 ml of pure alcohol.

**Abbreviations**: AO, alcohol‐only; AMED, alcohol mixed with energy drinks; AOCM, alcohol mixed with other caffeinated mixers; BAC, blood alcohol concentration; AUDIT‐C, Alcohol Use Disorder Identification Test.

One‐way analysis of variance showed there was a significant main effect of consumer type on the quantity of alcohol consumed, both predrinking (*F*(3, 368) = 4.839, *p* = .003, *η^2^p* = .013), on‐premise (*F*(3, 339) = 29.647, *p* ≤ .001, *η* ^2^p = .054), and overall consumption (*F*(3, 352) = 30.889, *p* < .001, *η* ^2^p = .062). In line with this, there were significant main effects on objective (*F*(3, 333) = 23.555, *p* < .001, *η* ^2^p = .041) and subjective intoxication (*F*(3, 351) = 26.363, *p* < .001, *η* ^2^p = .051). However, there were no significant main effects of consumption type on the time spent drinking that evening, either predrinking (*F*(3, 364) = .870, *p* = .457), on‐premise (*F*(3, 880) = .888, *p* = .447), or overall (*F*(3, 368) = .761, *p* = .517).

As can be seen in Table 2. Post hoc analysis revealed a consistent pattern of significant differences, with the AO consumer type drinking less alcohol and reporting feeling less intoxicated than all the alcohol–caffeine consumer types.

Specifically, the AO consumer type reported consuming significantly fewer alcohol units while predrinking compared to the AMED and mixed consumer types, but not the AOCM consumer type. Nonetheless, the AO consumer type did report consuming significantly fewer alcohol units while on‐premise and overall (predrinking plus on‐premise) in comparison to all other alcohol–caffeine consumer types.

Consistent with self‐reported alcohol consumption, the AO consumer type had a significantly lower BAC (minimum .03% BAC difference) and subjective intoxication (minimum 0.9 difference on 1–10 scale) than all alcohol–caffeine consumer types. The majority of post hoc differences (Hedge's g) fell within the medium to large (0.5 to > 1.0) effect size ranges (Lenhard & Lenhard, 2016). No significant differences were found between the alcohol–caffeine consumer types.

#### 3.1.2 Consumption on the night of the interview

To further differentiate whether there was a pharmacological effect of adding caffeine to alcohol, additional between‐subjects analyses were conducted among consumption groups based on a combination of consumer type and consumption on the night of the interview (AO, AMED, AOCM or mixed).

A significant main effect of consumption group on age (*F*(8, 224) = 8.156, *p* ≤ .001, *η* ^2^p = .06) and AUDIT‐C (*F*(8, 214) = 20.876, *p* ≤ .001, *η* ^2^p = .142) remained when consumer types were further differentiated by consumption on the night of the interview. Participants from the AO consumer type were significantly older when compared to the AMED and mixed consumer types, regardless of what they had consumed that evening. There were no significant differences in age between the AO and AOCM consumer type groups, or any other consumption groups. The alcohol‐only consumer type scored significantly lower on the AUDIT‐C when compared to all the alcohol–caffeine consumer groups (AMED consumer, AOCM consumer and mixed consumer groups), regardless of what they consumed that evening. There were no significant differences in gender (χ^2^(8, *N* = 893) = 14.40, *p* = .072), the percentage of smokers (χ^2^(8, *N* = 893) = 4.70, *p* = .789) or the number of cigarettes consumed on the night of the interview (*F*(8,212) = 1.728, *p* = .094) between any of the consumption groups.

One‐way analysis of variance showed that there was a significant main effect of consumption group on the quantity of alcohol consumed, both pre‐drinking (*F*(8, 216) = 3.036, *p* = .003, *η* ^2^p = .026), on‐premise (*F*(8, 208) = 11.601, *p* ≤ .001, *η* ^2^p = .092) and overall consumption (*F*(8, 213) = 13.400, *p* < .001, *η* ^2^p = .104). In line with this, there were significant main effects on objective (*F*(8, 211) = 10.564, *p* < .001, *η* ^2^p = .084) and subjective intoxication (*F*(8, 217) = 13.637, *p* < .001, *η* ^2^p = .091). However, there were no significant main effects of consumption group on the time spent drinking that evening, either predrinking (*F*(8, 577) = 0.866, *p* = .528), on‐premise (*F*(8, 215) = 1.220, *p* = .356), or overall (*F*(8, 884) = 1.234, *p* = .276).

Post hoc analysis showed that the AO consumer type consumed significantly less alcohol (both self‐report and BAC) and reported feeling less intoxicated than all of the alcohol–caffeine consumer types, regardless of what they had consumed that evening. Of importance, when comparing the different AO‐tonight groups, the AO consumer type consumed significantly less alcohol and reported feeling less intoxicated than all of the alcohol–caffeine consumer types. In addition, while there was a trend within consumer types toward increased alcohol consumption and subjective intoxication among alcohol–caffeine‐tonight groups compared to their respective AO‐tonight groups, these differences were not statistically significant.

**FIGURE 1:**
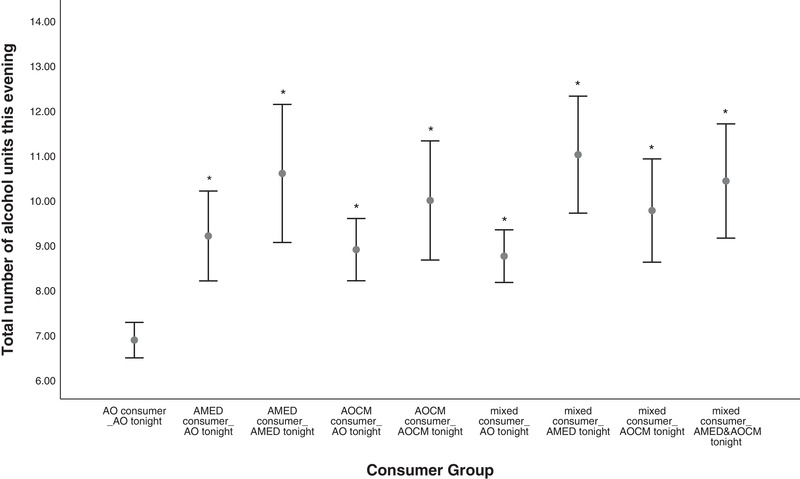
Between‐subjects comparison of consumer types by consumption on the night of the interview for total number of alcohol units consumed on the evening of testing. Notes: Means and 95% confidence intervals significantly different (*p < .05) to the AO consumer group. One alcohol unit = 8 g/10 ml of pure alcohol. AO, alcohol‐only; AMED, alcohol mixed with energy drinks; AOCM, alcohol mixed with other caffeinated mixers

**FIGURE 2:**
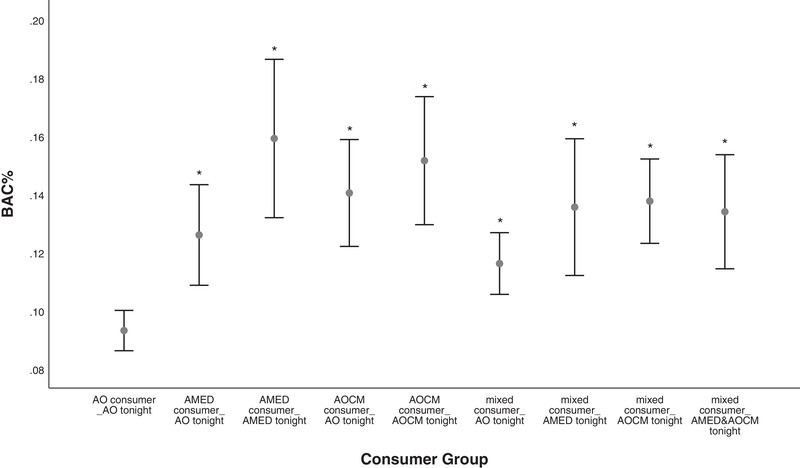
Between‐subjects comparison of consumer types by consumption on the night of the interview for BAC% on the evening of testing. Notes: Means and 95% confidence intervals significantly different (*p < .05) to the AO consumer group. AO, alcohol‐only; AMED, alcohol mixed with energy drinks; AOCM, alcohol mixed with other caffeinated mixers; BAC, blood alcohol concentration

**FIGURE 3:**
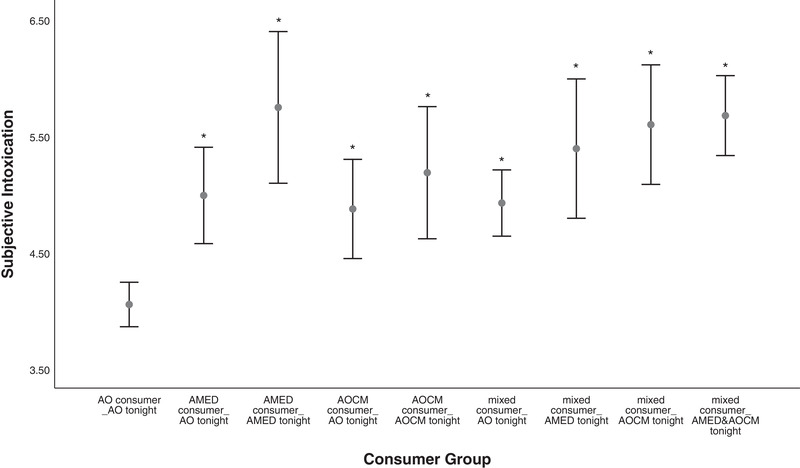
Between‐subjects comparison of consumer types by consumption on the night of the interview for subjective intoxication on the evening of testing. Notes: Means and 95% confidence intervals significantly different (*p < .05) to the AO consumer group. Subjective intoxication scale = 0–10. AO, alcohol‐only; AMED, alcohol mixed with energy drinks; AOCM, alcohol mixed with other caffeinated mixers

The majority of statistically significant post hoc differences (Hedge's g) fell within the medium to large (0.5 to >1.0) effect size ranges (Lenhard & Lenhard, 2016). A visual depiction of the means and 95% confidence intervals for total alcohol units (Figure 1), objective (Figure 2), and subjective intoxication (Figure 3) are included.

There were no significant differences in the number of energy drinks consumed, either predrinking (*F*(2,127) = .574, *p* = .565), on‐premise (*F*(2, 127) = .092, *p* = .912), or overall (*F*(2, 127) = .066, *p* = .937), between the AMED‐tonight consumer groups. Similarly, there were no significant differences in the number of caffeinated mixers consumed, either predrinking (*F*(2, 149) = .1.412, *p* = .247), on‐premise (*F*(2, 149) = .350, *p* = .705), or overall (*F*(2, 148) = .926, *p* = .399), between the AOCM‐tonight consumer groups. A summary of the data is presented in Table 3.

**TABLE 3:** Between‐subjects comparison of consumer types by consumption on the night of the interview

		AMED consumers		AOCM consumers		Mixed consumers			
		(*n* = 128)		(*n* = 135)		(*n* = 339)			
	AO consumers (*n* = 291) AO‐tonight (*n* = 291)	AO‐tonight (*n* = 85)	AMED‐tonight (*n* = 43)	AO‐tonight (*n* = 86)	AOCM‐tonight (*n* = 49)	AO‐tonight (*n* = 195)	AMED‐tonight (*n* = 41)	AOCM‐tonight (*n* = 57)	AMED & AOCM‐tonight (*n* = 46)
Age (years)	24.0 (4.1)	22.0 (2.9) [0.52]^a^	21.1 (2.0) [0.74]^a^	23.1 (5.0)	22.3 (4.2)	22.2 (3.9) [0.45]^a^	21.6 (3.6) [0.59]^a^	22.1 (3.3) [0.48]^a^	21.6 (4.0) [0.34]^a^
Gender (% female)	53.3 (± 5.7)	54.1 (± 10.6)	48.8 (± 14.9)	46.5 (± 10.5)	67.3 (± 13.1)	50.3 (± 7.1)	31.7 (± 14.3)	47.4 (± 12.9)	43.5 (± 14.3)
Student status (% students)	53.6 (± 5.7)	55.3 (± 10.6)	53.5 (± 14.9)	59.3 (± 10.4)	61.2 (± 13.6)	56.4 (± 6.9)	68.3 (± 14.3)	70.2 (± 11.9)	71.7 (± 13.1)
BAC%	0.09 (0.06)	0.13 (0.08) [0.62]^a^	0.16 (0.09) [1.08]^a^	0.14 (0.09) [0.74]^a^	0.15 (0.08) [0.95]^a^	0.12 (0.07) [0.47]^a^	0.14 (0.07) [0.82]^a^	0.14 (0.05) [0.85]^a^	0.13 (0.07) [0.65]^a^
Alcohol units predrinking	2.4 (2.8)	3.5 (3.3)	3.4 (3.1)	3.0 (2.3)	3.1 (2.7)	2.9 (3.2)	4.4 (2.9) [0.71]^a^	3.0 (3.6)	3.5 (3.2)
Alcohol units on‐premise	4.4 (2.3)	5.7 (3.1) [0.52]^a^	7.2 (3.6) [1.12]^a^	5.9 (3.0) [0.61]^a^	6.9 (4.5) [0.92]^a^	5.9 (2.7) [0.61]^a^	6.6 (3.4) [0.89]^a^	6.8 (3.7) [0.93]^a^	6.9 (3.7) [0.99]^a^
Total alcohol units	6.9 (3.4)	9.2 (4.6) [0.62]^a^	10.6 (5.0) [1.02]^a^	8.9 (3.2) [0.60]^a^	10.0 (4.6) [0.86]^a^	8.7 (4.2) [0.48]^a^	11.0 (4.1) [1.17]^a^	9.8 (4.3) [0.81]^a^	10.4 (4.3) [0.99^a^]
Subjective intoxication score	4.1 (1.7)	5.0 (1.9) [0.52]^a^	5.7 (2.1) [0.91]^a^	4.9 (2.0) [0.45]^a^	5.2 (2.0) [0.63]^a^	4.9 (2.0) [0.44]^a^	5.4 (1.9) [0.75]^a^	5.6 (1.9) [0.87]^a^	5.7 (1.2) [0.97]^a^
Total time predrinking	2:14 (1:31)	2:26 (1:25)	2:04 (1:40)	2:16 (1:33)	2:40 (1:45)	2:31 (1:46)	2:30 (1:27)	2:40 (2:17)	2:36 (1:31)
Total time on‐premise	3:36 (1:53)	3:11 (1:57)	3:44 (1:53)	3:40 (1:40)	3:07 (1:32)	3:20 (2:04)	3:14 (1:43)	3:22 (2:10)	3:40 (2:08)
Total drinking time	4:56 (2:28)	4:46 (2:22)	5:25 (2:18)	5:22 (1:57)	5:06 (2:31)	4:44 (2:32)	5:17 (1:55)	5:12 (2:20)	5:29 (2:20)
Energy drink unit: predrinking	–	–	0.6 (0.7)	–	–	–	0.5 (0.9)	–	0.4 (0.7)
Energy drink unit: on‐premise	–	–	1.1 (1.2)	–	–	–	1.2 (1.2)	–	1.2 (1.1)
Total energy drink units	–	–	1.7 (1.1)	–	–	–	1.7 (1.0)	–	1.6 (1.0)
Other caffeinated beverage unit: predrinking	–	–	–	–	0.7 (1.2)	–	–	0.9 (1.1)	0.5 (1.0)
Other caffeinated beverage unit: on‐premise	–	–	–	–	2.3 (1.9)	–	–	2.1 (1.8)	2.0 (1.8)
Total other caffeinated beverage units	–	–	–	–	3.1 (2.0)	–	–	2.9 (1.8)	2.5 (2.0)
Total AUDIT‐C Score (0–12)	6.2 (1.5)	7.8 (2.0) [0.98]^a^	8.0 (1.8) [1.17]^a^	7.2 (1.8) [0.64]^a^	7.9 (1.8) [1.10]^a^	7.8 (2.0) [0.93]^a^	8.1 (1.8) [1.23]^a^	7.8 (2.1) [0.99]^a^	7.5 (2.2) [0.81]^a^
Smoking (% yes)	23.7 (± 4.9)	27.1 (± 9.5)	37.2 (± 14.4)	17.4 (± 8.0)	30.6 (± 12.9)	27.7 (± 6.3)	24.4 (± 13.1)	28.1 (± 11.6)	30.4 (± 13.3)
Total number of cigarettes smoked	7.2 (4.1)	8.2 (2.2)	8.4 (1.8)	8.0 (4.1)	9.4 (4.3)	7.2 (3.5)	9.4 (2.8)	8.8 (2.0)	9.8 (4.2)

***Notes***: Mean and standard deviation (SD, between brackets) or percentages and 95% confidence interval (between brackets) are shown. Hedge's g effect size [between square brackets] where appropriate.

^a^ Significantly different (*p* < .05) to the AO consumer group. Subjective intoxication scale = 0–10, time = hours: minutes, one alcohol unit = 8 g/10 ml of pure alcohol, one energy drink unit = 80 mg caffeine, one caffeinated beverage unit = 80 mg caffeine.

**Abbreviations**: AO, alcohol‐only; AMED, alcohol mixed with energy drinks; AOCM, alcohol mixed with other caffeinated mixers; BAC, blood alcohol concentration; AUDIT‐C, Alcohol Use Disorder Identification Test.

#### 3.1.3 Relationship between objective and subjective intoxication

Comparing the association between objective (BAC) and subjective (0–10 scale) intoxication provides information on the relative level of subjective intoxication experienced for given objective intoxication levels. This enables potential masking effects due to caffeinated drink type to be examined. If a masking effect is present, then subjective intoxication scores may be lower for specific levels of objective intoxication (BAC) when compared to AO. However, these comparisons are based on consumption on the testing night, and therefore limited to between‐subject comparisons.

There was an overall significant positive correlation between BAC and scores of subjective intoxication (*r*(891) = .37, *p* ≤ .001).

**FIGURE 4:**
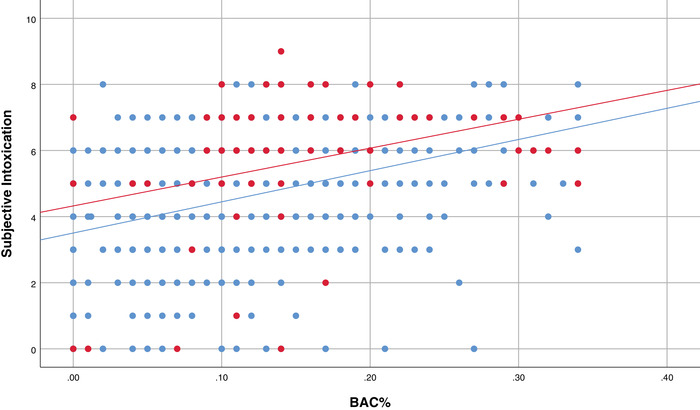
The relationship between objective intoxication (BAC%) and subjective intoxication for AO‐tonight and AMED‐tonight groups. Notes: Aggregated data is shown for participants from the AO‐tonight group (blue) and the AMED‐tonight group (red). Blue line represents the significant correlation between objective and subjective intoxication (R2 = 13.1%) for the AO‐tonight group. Red line represents the significant correlation between objective and subjective intoxication (R2 = 13.1%) for the AMED‐tonight group. Subjective intoxication scale = 0–10. AO, alcohol‐only; AMED, alcohol mixed with energy drinks; BAC, blood alcohol concentration

To investigate the possibility of a masking effect bivariate correlations were computed for those who consumed AO (*N* = 657) and those who consumed AMED (*N* = 84) on the night of the interview. Figure 4 shows that while there was a significant positive correlation between BAC and scores of subjective intoxication for both the AO‐tonight (*r*(655) = .36, *p* ≤ .001) and AMED‐tonight groups (*r*(82) = .36, *p* = .001), the difference between these correlations was not significant (*z* = 0.01, *p* = .99).

**FIGURE 5:**
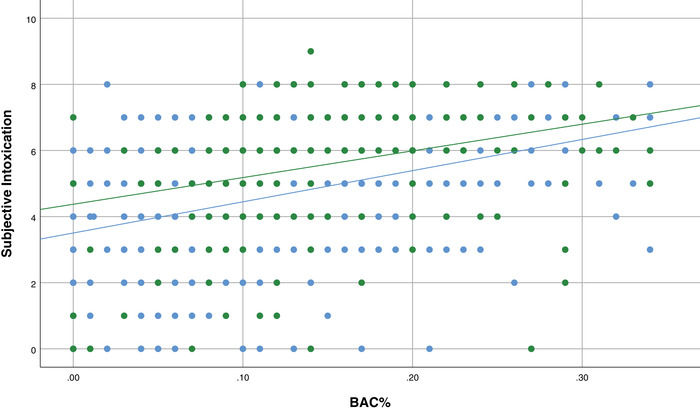
The relationship between objective intoxication (BAC%) and subjective intoxication for AO‐tonight and AC‐tonight groups. Notes: Aggregated data is shown for participants from the AO‐tonight group (blue) and the AC‐tonight group (green). Blue line represents the significant correlation between objective and subjective intoxication (R2 = 13.1%) for the AO‐tonight group. Green line represents the significant correlation between objective and subjective intoxication (R2 = 9.9%) for the AC‐tonight group. Subjective intoxication scale = 0–10. AO, alcohol‐only; AC, alcohol mixed with caffeine; BAC, blood alcohol concentration

Additional bivariate correlations were conducted for those who consumed AO (*N* = 657) and those who consumed alcohol with caffeine (AC, *N* = 236) in any form on the night of the interview (AMED‐tonight, AOCM‐tonight, AMED & AOCM‐tonight groups combined). In line with the above findings (Figure 5), there was also a significant positive correlation between BAC and scores of subjective intoxication for the AC‐tonight group (*r*(234) = 0.31, *p* ≤ .001), but the differences between the correlations for AO‐tonight and AC‐tonight were not significant (*z* = 0.01, *p* = .99). The overall subjective correlation plot lines for alcohol–caffeine beverages (AMED‐tonight = red, AC‐tonight = green) are higher than for AO‐tonight (blue).

**FIGURE 6:**
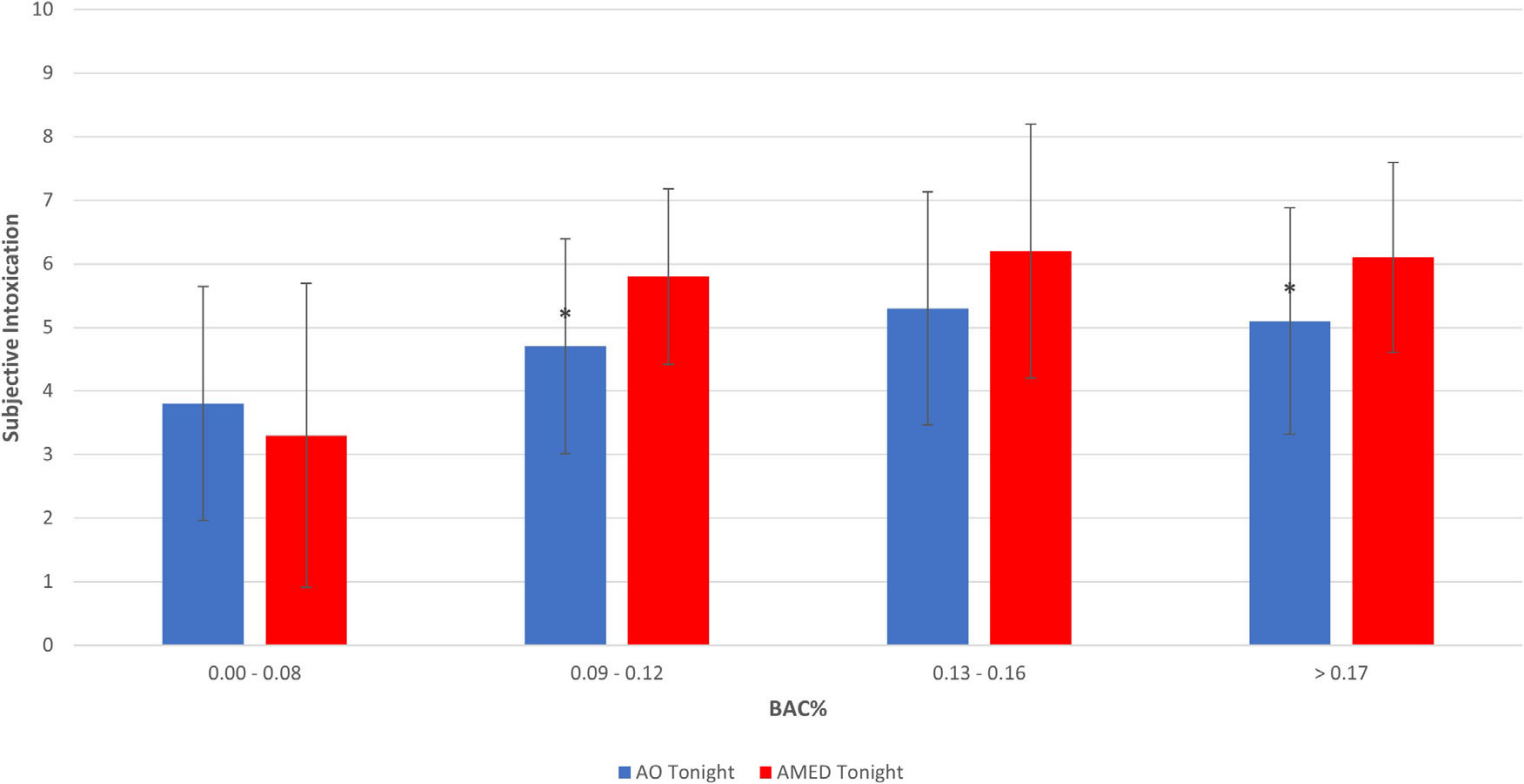
(Mean and standard deviation) subjective intoxication scores at different BAC% ranges for AO‐tonight and AMED‐tonight groups. Notes: *Significantly different (*p* < .05) between AO‐tonight and AMED‐tonight groups. Subjective intoxication scale = 0–10. AO, alcohol‐only; AMED, alcohol mixed with energy drinks; BAC, blood alcohol concentration

When considering mean differences at different BAC ranges, significantly higher subjective intoxication scores were observed in the 0.09%–0.12% (*t*(165) = −3.03, *p* = .003) and >0.17% (*t*(176) = −2.79, *p* = .006) ranges for the AMED‐tonight group compared to the AO‐tonight group. There were no significant differences at the 0.00%–0.08% (*t*(275) = 1.03, *p* = .30) and 0.13%–0.16% (*t*(117) = −1.86, *p* = .06) BAC ranges (Figure 6).

**FIGURE 7:**
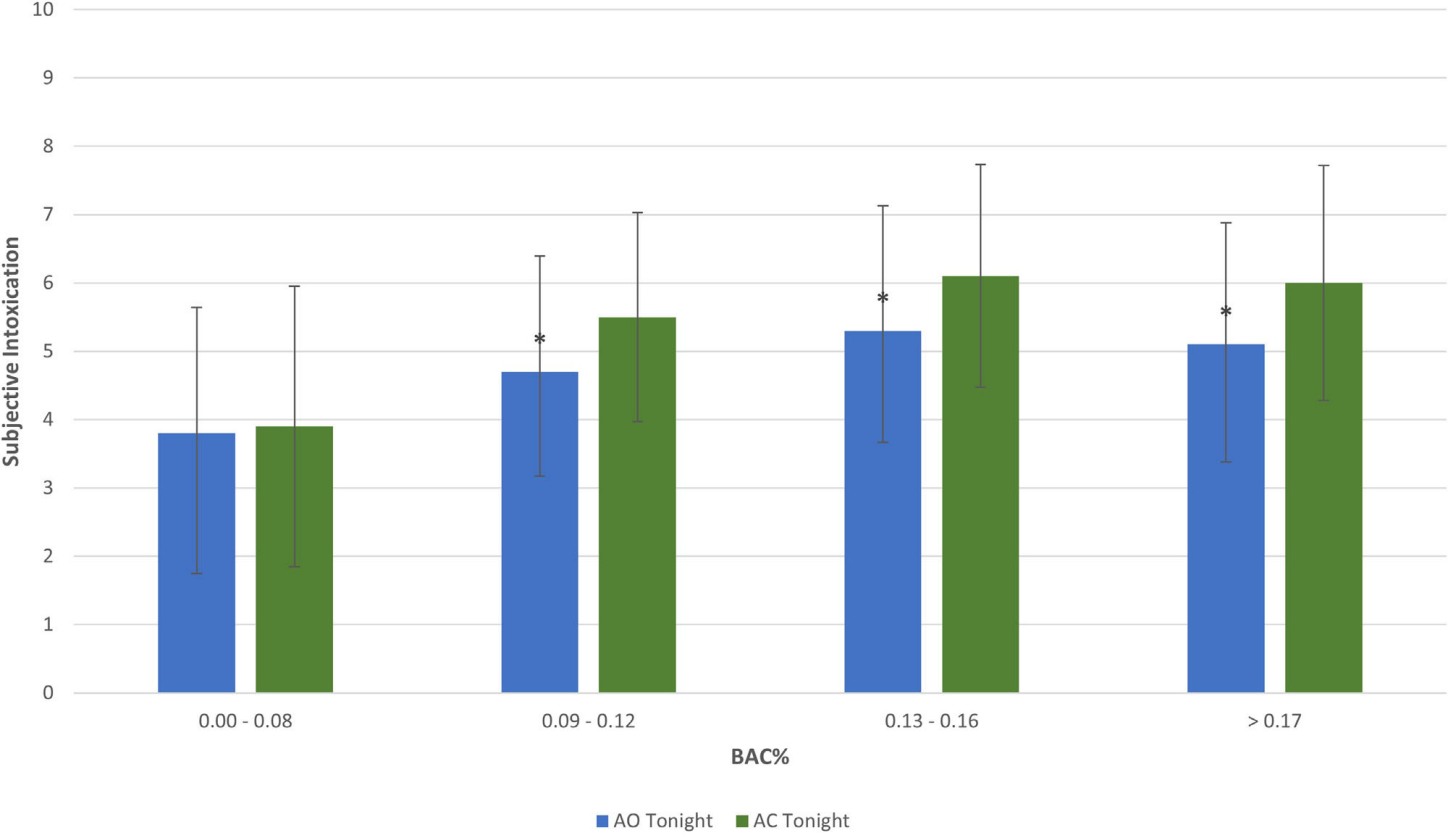
(Mean and standard deviation) subjective intoxication scores at different BAC% ranges for AO‐tonight and AC‐tonight groups. Notes: *Significantly different (*p* < .05) between AO‐tonight and AMED‐tonight groups. Subjective intoxication scale = 0–10. AO, alcohol‐only; AC, alcohol mixed with caffeine; BAC, blood alcohol concentration

Similarly, for the AC‐tonight group, significantly higher subjective intoxication scores were observed in the 0.09%–0.12% (*t*(223) = −3.28, *p* = .001), 0.13%–0.16% (*t*(142) = −2.50, *p* = .014), and >0.17% (*t*(223) = −3.63, *p* ≤ 0.001) BAC ranges compared to the AO‐tonight group (Figure 7). There were no significant differences at the 0.00%–0.08% BAC range (*t*(297) = −0.23, *p* = .82). For both AMED‐tonight and AC‐tonight, subjective intoxication ratings at given BAC ranges are similar or higher than the AO‐tonight group.

#### 3.1.4 Within‐subject comparisons

Of those who identified as AMED consumers (14.3%), 33.6% reported consuming AMED on the night of the interview and AO on other drinking occasions, with the remaining 66.4% reporting consuming AO on the night of the interview and AMED on other drinking occasions. There were no significant difference in the number of alcohol units consumed by the AMED‐tonight group (10.6 ± 5.0) on the night they were interviewed compared to AO (10.8 ± 5.2) drinking occasions (*t*(42) = 0.36, *p* = .723). However, the AMED‐other night group consumed significantly more alcohol units (9.2 ± 4.6) when they were interviewed (alcohol‐only) compared to reported consumption on AMED (7.9 ± 3.8) drinking occasions (*t*(84) = 3.08, *p* = .003, Cohen's *d* = 0.31). Although this fell within the small (0.2–0.5) effect size range (Lenhard & Lenhard, 2016).

Of those who identified as AOCM consumers (15.1%), 36.3% reported consuming AOCM on the night of the interview and AO on other drinking occasion, with the remaining 63.7% reporting consuming AO on the night of the interview and AOCM on other drinking occasions. No significant differences were found in the number of alcohol units reportedly consumed on AO and AOCM drinking occasions, for both the AOCM‐tonight (9.8 ± 5.2 vs. 10.0 ± 4.6, *t*(48) = –.25, *p* = .802) and AOCM‐other night (8.9 ± 3.2 vs. 8.3 ± 3.5, *t*(85) = 1.94, *p* = .056) consumer groups.

Of those participants who identified as mixed consumers (38.0%), 57.5% consumed AO on the night of the interview and AMED and AOCM on other drinking occasions, 12.1% consumed AMED on the night of the interview and AO and AOCM on other drinking occasions, 16.8% consumed AOCM on the night of the interview and AO and AMED on other drinking occasions, and 13.6% consumed both AMED & AOCM on the night of the interview on AO on other drinking occasions. A repeated measures ANOVA with Huynh–Feldt correction determined that there were no statistically significant differences in the amount of alcohol units consumed on AO, AMED, and AOCM drinking occasions for all mixed consumer consumption groups (alcohol‐only‐tonight: *F*(1.757, 340.821) = 2.735, *p* = .073, AMED‐tonight: *F*(1.501, 60.023) = 2.163, *p* = .136, AOCM‐tonight: *F*(1.335, 74.734) = 3.006, *p* = .075, AMED & AOCM‐tonight: *F*(1.227, 55.224) = 2.777, *p* = .094). A summary of the data is presented in Table 4.

**TABLE 4:** Within‐subject comparisons of alcohol–caffeine consumer types: Total number of alcohol units

	AO occasion	AMED occasion	AOCM occasion
AMED consumer—AO‐tonight	9.2 (4.6) [0.31]^a^	7.9 (3.8)	–
AMED consumer—AMED‐tonight	10.8 (5.2)	10.6 (5.0)	–
AOCM consumer—AO‐tonight	8.9 (3.2)	–	8.3 (3.5)
AOCM consumer—AOCM‐tonight	9.8 (5.2)	–	10.0 (4.6)
Mixed consumer—AO‐tonight	8.7 (4.2)	6.9 (3.4)	7.0 (3.3)
Mixed consumer—AMED‐tonight	10.9 (4.4)	11.0 (4.1)	10.1 (3.5)
Mixed consumer—AOCM‐tonight	9.2 (3.3)	8.4 (2.3)	9.8 (4.3)
Mixed consumer—AMED & AOCM‐tonight	10.0 (3.4)	9.7 (2.3)	9.5 (2.2)

***Notes***: Mean and standard deviation (SD, between brackets) are shown. Cohen's *d* effect size [between square brackets] where appropriate.

^a^ Significantly different (*p* < .05) between AO and AMED occasion. One alcohol unit = 8 g/10 ml of pure alcohol.

**Abbreviations**: AO, alcohol‐only; AMED, alcohol mixed with energy drinks; AOCM, alcohol mixed with other caffeinated mixers.

## 4 DISCUSSION

The present study aimed to determine whether there are any differences in alcohol consumption practices between those who consume AO and those who consume alcohol with different caffeinated mixers (AMED and AOCM). The findings confirm that alcohol–caffeine consumers consume more alcohol and have higher objective and subjective intoxication than those who consume AO. This was the case regardless of whether or not the alcohol–caffeine consumers mixed alcohol with caffeinated mixers or consumed AO on the night of the interview. In addition, no differences were found between alcohol–caffeine consumer groups.

These between‐subjects findings are consistent with previous survey‐based research (Verster et al., 2018, for a meta‐analysis) and on‐premise studies (Devilly et al., 2017; Lubman et al., 2014; Lubman et al., 2013; Miller et al., 2013; Pennay et al., 2015b; Thombs et al., 2009) that have found increased alcohol consumption among AMED consumers in comparison to AO consumers. While Verster et al. (2015) on‐premise study found no significant differences between AMED‐tonight and AO‐tonight consumers, they also had similar findings to the current study of no significant difference in alcohol consumption or subjective intoxication between the AMED‐tonight and AMED‐other night groups.

Between groups comparisons plotting subjective intoxication against BAC failed to reveal any masking effects (Verster et al., 2012) for either AMED or any caffeinated mixers, and in fact subjective intoxication scores were elevated for these groups at some BAC ranges compared to AO recorded at the time of assessment. This interesting finding should be further investigated in appropriately designed future studies, and while controlled within subject lab‐based comparisons are warranted, ethical limitations have currently restricted the upper BAC range of assessment to 0.12%, which only partially reflects average BACs recorded for groups in this study (0.09%–0.16%) and is well below the upper ranges found here and in other on‐premise studies that reflect actual consumption practices (Verster et al., 2015, 2018).

Given that objective and subjective intoxication was increased in the alcohol–caffeine consumer groups regardless of whether or not caffeinated mixers were consumed, or the type of caffeinated mixer consumed, suggests that the observed differences were caused by something other than the pharmacological interaction of caffeine with alcohol. A previous explanation proposed for increased alcohol consumption among AMED consumers has been a high risk‐taking personality (Verster et al., 2012). Individuals who are high‐risk takers are also more likely to exhibit certain other life‐style behaviors, such as increased frequency and amount of alcohol consumption, caffeine consumption, smoking, and recreational drug use; see Verster et al. (2018) for a review, although among AMED users risk behaviors may be reduced with AMED compared to AO drinking occasions (Newcombe et al., 2019). The findings from this study suggest that this could be extended to all alcohol–caffeine consumers. Thus, a personality with higher levels of risk‐taking behaviors may be the primary reason for increased alcohol use, and the coconsumption of caffeine with alcohol may just be one of many expressions of their lifestyle and personality. Further support for this notion comes from the finding that alcohol–caffeine consumer groups scored significantly higher on the AUDIT‐C compared to the AO group, increasing the possibility of damaging their health and the need for brief advice, intervention, or referral to alcohol services (World Health Organisation, 2001).

There are also alternative differences that may explain the increased alcohol consumption among alcohol–caffeine consumers compared to AO consumers. In line with previous AMED research (Azagba et al., 2013; Berger et al., 2011; Pennay et al., 2015a; Rutledge et al., 2016; Wells et al., 2013), the current study found that the alcohol–caffeine consumers were significantly younger than the AO consumers. Thus, younger consumers may be more attracted to drinking alcohol mixed with caffeine, particularly energy drinks, which may then dissipate with age as alcohol consumption decreases. This is supported by research (Office for National Statistics, 2018) indicating that binge drinking in the United Kingdom is at its highest between 16 and 24 years and decreases thereafter, while noting the average age of the AO consumers in this sample was 24 years.

To control for the many phenotypical differences that have been shown to exist between those who mix alcohol with caffeinated mixers and those who do not and to determine whether mixing alcohol with energy drinks or other caffeinated mixers has a different effect on overall alcohol consumption, within‐subject analyses were performed. In line with Verster et al. (2015), these findings revealed that AMED consumers drink the same, if not less alcohol on AMED occasions versus AO occasions. No significant difference was found in the amount of alcohol consumed by AOCM consumers on AOCM and AO occasions. Neither were there any significant differences in the amount of alcohol reportedly consumed by mixed consumers on AMED, AOCM, and AO occasions. Thus, mixing alcohol with caffeine in any form did not increase total alcohol consumption.

These findings are significant given that this is the first on‐premise study to differentiate alcohol–caffeine consumers using both between and within‐subject comparisons. In addition, the present study has also illustrated that while alcohol–caffeine consumption is a popular consumption choice, with the majority of participants (67.4%) identifying as alcohol–caffeine consumers in some form (mixed consumers 38%, AOCM consumers 15.1%, AMED consumers 14.3%), consumption is less frequent than AO with only 26.5% reporting alcohol–caffeine consumption on the night of the interview.

Collection of alcohol consumption in an ecologically valid setting meant participant responses were not as limited by retrospective recall or ethical limitations. However, it should be noted that the within‐subject comparison included the on‐premise evening versus another occasion in the past, thus it could be claimed that this measure may have been affected by recall bias or alcohol related amnestic effects. It must also be considered that participants responses may have been impacted by the amount of alcohol consumed, with some people exaggerating or understating their level of intoxication. To overcome this, the interview was kept short, consisted of simple questions and a level of quality control was applied by the research assistant fact‐checking extreme responses as suggested by Miller et al. (2017).

A limitation of on‐premise methodology in general is that it only provides a snapshot of the drinking occasion and fails to provide event level information on the patterns of use, motivations and consequences of consumption. Future research could adopt prospective longitudinal designs to examine the temporal relationship between alcohol–caffeine consumption. However, these are expensive and time consuming.

A further limitation of the current study is that it did not consider the impact of dietary mixers on alcohol consumption. Indeed, previous research (Rossheim & Thombs, 2011) has demonstrated that caffeine's effect on intoxication may be most pronounced when mixers are artificially sweetened. To investigate this further, future research could differentiate between diet or nondietary caffeinated mixers using the applied methodology.

Another methodological limitation of the current study is that despite asking participants about their smoking and drug use on the night of the interview no objective testing was conducted. Therefore, it may have been possible that drug users were included in the sample or that participants had smoked within 15 min prior to the alcohol breath test. However, the time taken to explain the study and gain informed consent would likely have exceeded this timeframe. Future studies should therefore endeavor to administer objective tests of smoking and drug use, although this may impact on participants willingness to engage with the study.

As our study was the first to assess alcohol consumption among different alcohol–caffeine consumers, using both between and within‐subject comparisons, additional research is needed to replicate these findings in other samples. Indeed, the external validity of our results is restricted to a predominantly student population in one UK city (Bristol). Future research should be conducted in other urban and rural areas in the United Kingdom as well as in other countries.

## 5 CONCLUSION

In summary, the findings of this on‐premise study demonstrate that increased alcohol consumption is not unique to AMED consumers only, but extends to all alcohol–caffeine consumers. However, within‐subject findings demonstrate that mixing alcohol with caffeine in any form has no impact on overall alcohol consumption. This provides evidence that alcohol–caffeine use may indicate a personality predisposition to higher risk‐taking behaviors that results in the engagement with caffeinated mixer user. Thus, alcohol–caffeine use, and not just AMED use, may be a useful indicator for healthcare practitioners to identify individuals who may benefit the most from risk and harm reduction strategies for excessive alcohol consumption per se.

## CONFLICTS OF INTEREST

The funder had no role in the design of the study; in the collection, analyses, or interpretation of data; in the writing of the manuscript, or in the decision to publish the results.

S.J.J has undertaken sponsored research for Pfizer, AstraZeneca, Merck, Gilead, Novartis, Roche, Red Bull GmbH, the Department for Transport, and Road Safety Trust. Over the past 3 years, J.C.V has acted as a consultant for More Labs, Red Bull, Sen‐Jam Pharmaceutical, Toast!, Tomo, and ZBiotics. C.A. has undertaken sponsored research, or provided consultancy, for a number of companies and organizations including Airbus Group Industries, Astra, British Aerospace/Bae Systems, Civil Aviation Authority, Duphar, Farm Italia Carlo Erba, Ford Motor Company, ICI, Innovate UK, Janssen, LERS Synthélabo, Lilly, Lorex/Searle, More Labs, UK Ministry of Defense, Quest International, Red Bull GmbH, Rhone‐Poulenc Rorer, Sanofi Aventis and Vital Beverages.

### PEER REVIEW

The peer review history for this article is available at https://publons.com/publon/10.1002/brb3.2445
